# Assessments of the Ecological and Health Risks of Potentially Toxic Metals in the Topsoils of Different Land Uses: A Case Study in Peninsular Malaysia

**DOI:** 10.3390/biology11010002

**Published:** 2021-12-21

**Authors:** Chee Kong Yap, Weiyun Chew, Khalid Awadh Al-Mutairi, Rosimah Nulit, Mohd. Hafiz Ibrahim, Koe Wei Wong, Alireza Riyahi Bakhtiari, Moslem Sharifinia, Mohamad Saupi Ismail, Wah June Leong, Wen Siang Tan, Wan Hee Cheng, Hideo Okamura, Chen Feng You, Salman Abdo Al-Shami

**Affiliations:** 1 Department of Biology, Faculty of Science, Universiti Putra Malaysia (UPM), Serdang 43400, Malaysia; chewweiyun@gmail.com (W.C.); rosimahn@upm.edu.my (R.N.); mhafiz_ibrahim@upm.edu.my (M.H.I.); wongkoewei@gmail.com (K.W.W.); 2 Department of Biology, Faculty of Science, University of Tabuk, Tabuk P.O. Box 741, Saudi Arabia; kmutairi@ut.edu.sa; 3 Department of Environmental Sciences, Faculty of Natural Resources and Marine Sciences, Tarbiat Modares University, Noor 46417-76489, Iran; riahi@modares.ac.ir; 4 Shrimp Research Center, Iranian Fisheries Science Research Institute, Agricultural Research, Education and Extension Organization (AREEO), Bushehr 75169-89177, Iran; moslem.sharifinia@yahoo.com; 5 Fisheries Research Institute, Batu Maung 11960, Pulau Pinang, Malaysia; saupi@rocketmail.com; 6 Department of Mathematics and Statistics, Faculty of Science, Universiti Putra Malaysia (UPM), Serdang 43400, Malaysia; leongwj@upm.edu.my; 7 Department of Microbiology, Faculty of Biotechnology and Biomolecular Sciences, Universiti Putra Malaysia (UPM), Serdang 43400, Malaysia; wstan@upm.edu.my; 8 Laboratory of Vaccines and Biomolecules, Institute of Bioscience, Universiti Putra Malaysia (UPM), Serdang 43400, Malaysia; 9 Faculty of Health and Life Sciences, Inti International University, Persiaran Perdana BBN, Seremban 71800, Malaysia; wanhee.cheng@newinti.edu.my; 10 Graduate School of Maritime Sciences, Faculty of Maritime Sciences, Kobe University, Kobe 658-0022, Japan; okamurah@maritime.kobe-u.ac.jp; 11 Department of Earth Sciences, National Cheng-Kung University, No 1, University Road, Tainan City 701, Taiwan; cfy20@mail.ncku.edu.tw; 12 Indian River Research and Education Center, IFAS, University of Florida, Fort Pierce, FL 34945, USA; alshami200@gmail.com

**Keywords:** ecological risk, potentially toxic metals, peninsular Malaysia, topsoils

## Abstract

**Simple Summary:**

This study reported the ecological risks and human health risk assessments of five potentially toxic metals in the topsoils of six land uses in Peninsular Malaysia. It was found that industry, landfill, rubbish heap, and mining areas were categorized as “very high ecological risk”. The land uses of industry, landfill and rubbish heap were found to have higher hazard quotient values for the three pathways of the five metals for children and adults, when compared to the mining, plantation, and residential areas. The values for both the non-carcinogenic (Cd, Cu, Ni, and Zn), and carcinogenic risks for inhalation (Cd and Ni) obtained for children and adults in this study showed no harmful health effects on their health. However, of public concern, the hazard index, for Pb of children at the landfill and the rubbish heap showed non-carcinogenic risk for children. Therefore, children need to be taken care from public standpoint. They should be advised not to play in the topsoils near industry, landfill and rubbish heap areas. The present findings are important for the environmental management of potentially toxic metals especially in the land uses of industry, landfill and rubbish heap in Peninsular Malaysia.

**Abstract:**

Human activities due to different land uses are being studied widely in many countries. This study aimed to determine the ecological risks and human health risk assessments (HHRA) of Cd, Pb, Ni, Cu, and Zn in the topsoils of six land uses in Peninsular Malaysia. The ranges of the potentially toxic metals (PTMs) in the soils (mg/kg, dry weight) of this study were 0.24–12.43 for Cd (mean: 1.94), 4.66–2363 for Cu (mean: 228), 2576–116,344 for Fe (mean: 32,618), 2.38–75.67 for Ni (mean: 16.04), 7.22–969 for Pb (mean: 115) and 11.03–3820 for Zn (mean: 512). For the ecological risk assessments, the potential ecological risk index (PERI) for single metals indicated that the severity of pollution of the five metals decreased in the following sequence: Cd > Cu > Pb > Zn > Ni. It was found that industry, landfill, rubbish heap, and mining areas were categorized as “very high ecological risk”. For HHRA, the land uses of industry, landfill and rubbish heap were found to have higher hazard quotient (HQ) values for the three pathways (with the order: ingestion > dermal contact > inhalation ingestion) of the five metals for children and adults, when compared to the mining, plantation, and residential areas. The values for both the non-carcinogenic (Cd, Cu, Ni, and Zn), and carcinogenic risks (CR) for inhalation (Cd and Ni) obtained for children and adults in this study showed no serious adverse health impacts on their health. However, of public concern, the hazard index (HI), for Pb of children at the landfill (L-3) and the rubbish heap (RH-3) sites exceeded 1.0, indicating non-carcinogenic risk (NCR) for children. Therefore, these PERI and HHRA results provided fundamental data for PTMs pollution mitigation and environmental management in areas of different land uses in Peninsular Malaysia.

## 1. Introduction

Human activities due to different land uses such as landfills, vehicles, mining, industries, residential, agricultural plantations, and city garbage disposal are usually related to soil-heavy metal pollutions [1,2,3]. All these activities can contribute to the anthropogenic heavy metal pollution in urban areas [4,5,6,7].

The pollution of environments by heavy metals is a global issue, as rapid industrialization worldwide has significantly contributed to the release of theoretically potentially toxic metals (PTMs) into soils and water [8,9]. The elevated levels of PTMs in the topsoils of the different land uses may cause the weakening of the soil biological system, undermine human wellbeing, and create many environmental issues. Therefore, PTMs pollution in topsoils is of increasing concern from the environmental management perspective.

The rapid development in Malaysia has increased the output of anthropogenic PTMs inputs into its environment [10]. In Peninsular Malaysia, numerous studies have reported the occurrences of PTMs pollution in coastal areas, estuarine rivers, mangroves, urban areas, lakes, etc. due to increasing urban activities [11,12,13,14,15,16,17]. However, limited studies have focused on terrestrial pollution in Malaysia.

Long term exposure to PTMs pollutants potentially poses harmful effects on human health [18]. In recent years, soils of different land uses (such as residential urban areas) have been studied as diagnostic tools of environmental conditions that influence human health [19,20,21,22]. Several studies have reported on human health risk (HHR) of PTMs pollution in soils and road dust [23,24]. The urban soil polluted by PTMs could enter the human body by three different pathways: ingestion, inhalation, and dermal contact [1,21,25,26,27]. Furthermore, numerous investigations have revealed the negative impacts of elevated metals on human health [18,23].

Based on the recent literature [2,27,28,29], the number of papers published on human health risk assessment (HHRA) of PTMs in soils from different areas in the world will continue to increase in the future as they are closely related to the public health of both children and adults. The HHRA of PTMs in soils collected from different land uses had been reported mainly from China [30,31,32]. However, HHR due to metal contamination in different land uses from Malaysia have not been studied intensively. Therefore, in the present study, various sampling sites including landfill, industrial, residential, rubbish heap, and abandoned mining and plantation areas in Peninsular Malaysia were investigated. The specific objectives of this study were (1) to determine the concentrations of PTMs (Cd, Cu, Zn, Pb, and Ni), and (2) to assess the potential ecological risks and the HHRA of PTMs, in topsoils collected from different land uses in Peninsular Malaysia.

## 2. Materials and Methods

### 2.1. Sampling Site Descriptions and Soil Collection

**Figure 1:**
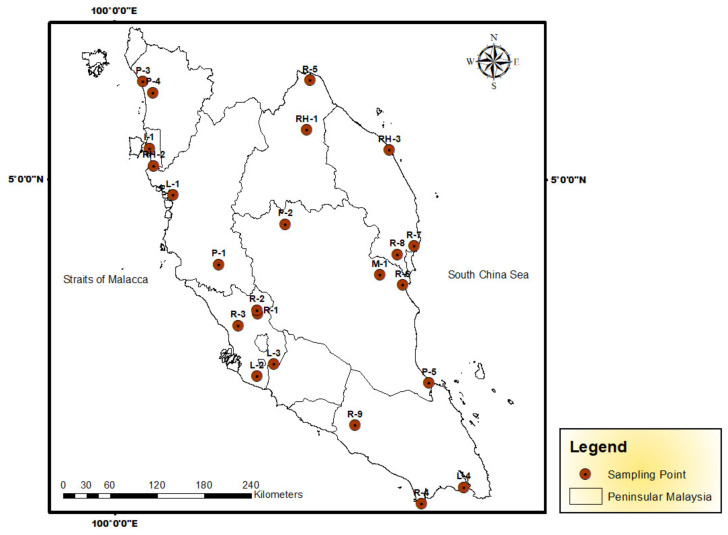
Sampling sites in Peninsular Malaysia (Numbers of sampling sites are described in Table 1).

Samplings of topsoils (0–10 cm) were done at 23 sites from 8 June 2011 to 17 January 2012, in Peninsular Malaysia (Figure 1). A list of the sampling sites is presented in Table 1. At each sampling site, three to five subsamples were collected from the topsoil (0–10 cm) using a stainless steel shovel. About 2 kg of topsoil were collected from each site. These sub-samples were thoroughly mixed to form a composite sample. The samples were placed in zipped-lock polyethylene bags and transferred to the laboratory. Upon reaching the laboratory, the soil samples were oven-dried at 80 °C for 72 h and passed through a 2 mm nylon sieve to remove external particle materials. The dried soils were passed through 63 µm sieves to dissolve in the acid digestion of the soil particles completely for heavy metal analysis. This was because the highest metal concentrations such as Pb was in the smallest fraction analyzed (<63 μm) for the assessment of incidental ingestion [33,34].

Locations’ characteristics were observed during the time of sampling. Sampling sites’ characteristics were divided into 6 groups: residential area, plantation area, landfill area, rubbish heaps, industrial area, and mining area.

In this study, topsoils were collected from Juru (I-1), a known polluted active industrialized area in Juru Industrial Estate [6,7,35,36,37,38]. All reported studies showed heavy metal pollution in Juru river basin, whether in the river, estuary or offshore sections. The industrial area’s sampling sites were surrounded by heavy industrial activities. Therefore, the land use in Juru is mainly industrial.

Sg. Lembing (Kuantan) is an abandoned tin mining site which has caused environmental concerns because waste materials from the abandoned mines may pollute the river and groundwater with harmful materials such as As, Fe, Cu, Pb, Ni, and Zn. These metal elevations can affect the water quality level in the stream [39]. The Sg. Lembing (M-1) site was located within an abandoned tin mining area, surrounded by dense trees and close to a green field. These two sites were considered as reference sites, and therefore there was only one sampling site for each of them in Juru and Sg. Lembing.

For the plantation area, the five sites were sampled in the plantation itself, either from the paddy field or the palm oil plantation. Plantation areas have low vehicular frequency. The Kg. Ayer Hitam (P-1) site was located within the palm oil plantation. The Perah (P-2) site was located by a road beside a shop building heavily surrounded by dense trees. The Alor Setar (P-3) site was located within a paddy field, close to a greenfield and a road. The Pendang (P-4) site was located at the side of a water canal surrounded by paddy field. The Tg. Gemok (P-5) site was located at the side of a farm/orchard close to a housing area.

For the landfill area, the four sites were located in close vicinity to the landfill sites. The Matang (L-1) open landfill site was located within the landfill facility site (about 300 m × 300 m), close to the leachate site about 200 m away. The total area of the Matang landfill was 12 ha, and it was classified as an improved anaerobic landfill, which was operated for over 14 years. [40]. The Sepang (L-2) landfill site was located within the landfill facility site (about 400 m × 400 m), with open green fields and dense trees in the vicinity. This L-2 site was located near the Tanjung Dua Belas Sanitary open landfill in Sepang, and it was operated for more than 10 years. The Sg. Kembong (L-3) open landfill site was located at the side of a landfill facility (>500 m × 500 m) beside a river. It was opened in 1989 and closed in 2010. The Sg. Kembong landfill was classified as a Type I non-sanitary landfill [41]. The Tanjung Langsat (L-4) open landfill site was located by a road of a landfill facility (about 500 m × 300 m), which was surrounded by dense trees. It is located in Pasir Gudang, receiving mainly municipal solid waste. The L-4 landfill covered about 50 acres, half of which was for the disposal of wastes, and the remaining was for treatment and maintenance facilities. It was operated for more than 10 years [42].

For the residential area, the nine sites have observable residential housing at proximity. The Kg. Bukit Chandang (R-1) site was located within a residential area and close to dense trees, about 100 m from a highway. The Kg. Bkt. Rasa (R-2) site was located at the green field of a residential area, and about 50 m away from a highway. The Ijok (R-3) site was located within the palm oil plantation close to a housing area. The Tanjung Piai (R-4) site was located within a housing area with open fields, just about 50 m from the seaside. The Kota Bahru (R-5) site was located within a green field, close to a housing area and dense trees. The Kuantan (R-6) site was located at the side of a dense tree area, close to a housing area. The Chukai/Kemaman (R-7) site was located within a housing area, close to dense trees. The Cheneh (R-8) site was located in dense trees close (20 m) to a housing area. The Pagoh (R-9) site was located within a dense tree area, close (about 15 m) to a housing area, a river, and a water treatment facility (15 m).

For the rubbish heap area, the three sites were located with observable municipal waste dumping, legally or illegally. The Kuala Krai (RH-1) site was located near a shop building beside a road, close to a palm oil plantation and green field. The Nibong Tebal (RH-2) site was located on a small road between the housing area and dense trees. The Kuala Terengganu (RH-3) site was located within a green field surrounded by a commercial area.

### 2.2. Metal Analysis

#### 2.2.1. Acid Digestions for Topsoil

The direct aqua-regia, which is a wet digestion method, was used to digest the soil samples. A total of 0.50 g of dried topsoilsamples was placed in a digestion tube (3 replicates). The aqua regia is a mixture of nitric acid (HNO_3_; AnalaR grade, BDH 69%) and perchloric acid (HClO_3_; AnalaR grade, BDH 60–70%), in a ratio of 4:1. The digestion tube was heated at 40 °C for an hour and then at 140 °C for the next 2–3 h on a digestion block [43]. At the end of the 4th hour, the brownish fume stop emitting, indicating the end of the digestion. Then, the digested solution was added-up to 40 mL with distilled water. Whatman No.1 filter paper was used to filter the solution. Acid-washed polyethylene bottle was used to store the solution [43]. The solution was analyzed using an air-acetylene flame atomic absorption spectrophotometer (FAAS, Perkin Elmer Model AAnalyst 800; Perkin Elmer LLC, Branford, CT, USA).

#### 2.2.2. Quality Control for Heavy Metal Analysis

All glassware and equipment used were acid-washed to avoid external contamination. Procedural blanks and quality control samples made from the standard solution for each metal were analyzed along with the digested samples. These standard solutions were analyzed after every 5–10 samples in order to check for the accuracy of the analyzed samples The accuracy of the methods for the analysis of Cd, Cu, Fe, Ni, Pb, and Zn was verified with the Certified Reference Materials (CRM) of NSC DC73319 Soil China, MESS - 3 NRC, TH-1 Sediment Canada, SRM 1547, and IAEA Soil-5. Comparisons of the percentage recoveries for the six metals between the certified values of the CRM and the measured concentrations are presented in Table 2. The recoveries were 102–156% for Cd, 85.0–93.1% for Cu, 96.6–106% for Fe, 102–112% for Ni, 99.8–116 for Pb, and 82.8–115% for Zn (Table 2). The detection limits of the FAAS for Cd, Cu, Fe, Ni, Pb, and Zn were 0.009, 0.010, 0.010, 0.010, 0.009, and 0.007 mg/L, respectively.

### 2.3. Data Treatment

#### 2.3.1. Geoaccumulation Index

Geoaccumulation index (Igeo) has been proved as an effective method for soil and sediment heavy metal contamination evaluation [44,45,46]. The geoaccumulation index (I_geo_) was used to determine the degree of metal pollution in the area. The calculation of I_geo_ was based on Equation (1) [47].

(1) $$I_{\mathrm{geo}}=\log_{2}\frac{\mathrm{Sample}}{1.5\times \mathrm{Background}}$$

where ample is the concentration measured while background is the background concentration in the earth’s upper continental crust (UCC). The UCC values were taken from Wedepohl [48], namely Cd (0.10 mg/kg), Cu (25.0 mg/kg), Fe (43,000 mg/kg), Ni (56.0 mg/kg), Pb (15.0 mg/kg), and Zn (65.0 mg/kg), because there is no verified information available on the background concentrations for soils in Peninsular Malaysia.

The value (1.5) is the correction factor to mitigate the lithogenic effluents. There are six established classifications of pollution: “practically unpolluted” (<0), “unpolluted” (0–1), “moderately polluted” (1–2), “moderately polluted to strongly polluted” (2–3), “strongly polluted” (3–4), “strongly to very strongly polluted” (4–5), and “very strongly polluted” (>5) [47].

The use of Igeo in the soils has been widely reported in literature recently, including Guangzhou-Foshan urban soils of South China [45], Anshan industrial city (Northeast China) [21], a municipal solid waste dump in Uyo (Nigeria) [49], ithallium mining area of southwest Guizhou (China) [50], Harran Plain (Turkey) [51], trailer park in Nigeria [28], Houzhai River Watershed of Guizhou Province (China) [46], wheat cultivated and natural soils of pastoral lands in the Bai Cheng Region (Xinjiang, China) [52], Zhundong mining area in Xinjiang [53], a Ramsar site (Deepor Beel) (Guwahati, India) [54], Panzhihua (China) [55], and the city of Lisbon (Portugal) [56].

#### 2.3.2. Contamination Factor

The calculation of contamination factor (CF) was based on the pollution of a single metal factor in Equation (2).

(2) $$\mathrm{CF}=\frac{C_{s}}{C_{B}}$$

where C_s_ is the concentration of PTM in topsoil. C_B_ is the background value of each PTM in the topsoil. The present study used the earth’s UCC values provided by Wedepohl [48] as background values.

#### 2.3.3. Pollution Load Index (PLI)

The pollution load index (PLI), proposed by Tomlinson et al. [57], was calculated using Equation (3).

PLI = (CF_1_ × CF_2_ × CF_3_ … × CF_N_)^1/N^ (3)

where N is the number of metals studied, and CF is the contamination factor (Cf) calculated as described in equation 3. The PLI gives an estimation of the metal contamination status, and the necessary action that should be taken. A PLI < 1 denotes perfection, PLI = 1 indicates that only baseline levels of pollutants are present, and PLI > 1 indicates deterioration of site quality [57].

According to contamination degree, the PLI is classified as “unpolluted” (PL ≤ 1), “unpolluted to moderately polluted” (1 < PL ≤ 2), “moderately polluted” (2 < PLI ≤ 3), “moderately to highly polluted” (3 < PLI ≤ 4), “highly polluted” (4 < PLI ≤ 5), and “very highly polluted” (PLI > 5) [19,21,58,59,60].

The use of PLI in the soils has been widely reported in literature recently such as in Industrial area of Hyderabad (India) [61], Anshan industrial city (Northeast China) [21], urban soils of Bangladesh [59], a municipal solid waste dump in Uyo (Nigeria) [49], paddy soils of Omor Rice Field, Nigeria [62], Kpone landfill site (Ghana) [63], a Ramsar site (Deepor Beel) (Guwahati, India) [54], and Southern Yunnan Province (China) [64].

#### 2.3.4. Ecological Risk Index

The calculation of ecological risk (Er), which is the potential ecological risk of a single element, was calculated based on Equation (4).

(4) $$\mathrm{ER}=T_{R}\times \mathrm{Cf}$$

where T_R_ is the toxic response factor of a single element. The T_R_ values used in the present study are Cd = 30.0, Cu = 5.00, Ni = 5.00, Pb = 5.00, and Zn = 1.00 [65]. According to Hakanson [65], 5 classifications for the Er are “low potential ecological risk” (ER < 40), “moderate potential ecological risk” (40 ≤ ER < 80), “considerable potential ecological risk” (80 ≤ ER < 160), “high potential ecological risk” (160 ≤ ER < 320), and “very high ecological risk” (ER ≥ 320).

#### 2.3.5. Potential Ecological Risk Index

Potential ecological risk index (PERI) was used to determine the potential risk of the PTMs in the topsoil to the ecology. This PERI was proposed by Hakanson [65]. The summation of all the ER values from each PTM give rise to the PERI value, which was calculated based on Equation (5).

(5) PERI=∑ER

According to Hakanson [65], 4 classifications for PERI values are “low ecological risk” (PERI < 150), “moderate ecological risk” (150 ≤ PERI < 300), “considerable ecological risk” (300 ≤ PERI < 600), and “very high ecological risk” (PERI ≥ 600).

This index is a relatively rapid, simple, and standard method for assessing the potential ecological risk level of PTEs in soils or sediments to the environment [44,45,50]. Although this method is based on the principle of sedimentology and aquatic ecosystem, it has been used in the soil pollution evaluation [66,67].

The use of ER and PERI has been widely reported in literature recently such as in polluted farmland soils from China [67], Guangzhou-Foshan urban soils of South China [45], Recife metropolitan region in Brazil [68], industrial area of Hyderabad (India) [61], iron ore mining in Pahang, Malaysia [69], in Anshan industrial city (Northeast China) [21], a municipal solid waste dump in Uyo (Nigeria) [62], paddy soils of Omor Rice Field, Nigeria [62], thallium mining area of southwest Guizhou (China) [50], Harran Plain (Turkey) [51], trailer park in Nigeria [28], a copper smelter in Khatoon Abad (Iran) (Nematollahi et al. 2020), Houzhai River Watershed of Guizhou Province (China) [46], Kpone landfill site (Ghana) [63], wheat cultivated and natural soils of pastoral lands in the Bai Cheng Region (Xinjiang, China) [52], Zhundong mining area in Xinjiang [53], a Ramsar site (Deepor Beel) (Guwahati, India) [54], Panzhihua (China) [55], Southern Yunnan Province (China) [64], and the city of Lisbon (Portugal) [56].

## 3. Human Health Risk Assessment

Human health risk assessment (HHRA) of topsoils is generally utilized to measure both carcinogenic risk (CR) and non-carcinogenic risk (NCR) to humans by means of three exposure pathways, namely ingestion, inhalation, and dermal contact. The methodology utilized for the HHRA depended on the guidelines and Exposure Factors Handbook of US Environmental Protection Agency [70,71,72,73]. The average daily doses (ADDs) (mg/kg day) of PTMs through ingestion (ADD_ing_), inhalation (ADD_inh_) and dermal contact (ADD_der_) for both children and adults were calculated by using Equations (6)–(8) as follows:

(6) $${\mathrm{ADD}}_{\mathrm{ing}}=C_{\mathrm{soil}}\left(\frac{\mathrm{IngR}\times \mathrm{EF}\times \mathrm{ED}}{\mathrm{BW}\times \mathrm{AT}}\right)\times 10^{-6}$$

(7) $${\mathrm{ADD}}_{\mathrm{inh}}=C_{\mathrm{soil}}\left(\frac{\mathrm{InghR}\times \mathrm{EF}\times \mathrm{ED}}{\mathrm{PEF}\times \mathrm{BW}\times \mathrm{AT}}\right)$$

(8) $${\mathrm{ADD}}_{\mathrm{der}}=C_{\mathrm{soil}}\left(\frac{\mathrm{SA}\times \mathrm{AF}\times \mathrm{ABS}\times \mathrm{EF}\times \mathrm{ED}}{\mathrm{BW}\times \mathrm{AT}}\right)\times 10^{-6}$$

where ADD_ing_, ADD_inh_ and ADD_der_ are the daily amounts of exposure to metals (mg/kg day) through ingestion, inhalation and dermal contact, respectively. In this study, NCR of PTMs was assessed by using the hazard quotient (HQ) and hazard index (HI), while the carcinogenic effects by the carcinogenic risk (CR) methods [20,21]. The definition, exposure factors and reference values used to estimate the intake values and health risks of PTMs in topsoils collected from Peninsular Malaysia are presented in Table 3.

The HQ is the proportion of the ADD of a metal to its reference dose (RfD) for the similar exposure pathway(s) [72]. The RfD (mg/kg day) is the maximum daily dose of metal from a particular exposure pathway, for both children and adults, that is accepted not to prompt a considerable risk of harmful effects to sensitive individuals during a lifetime. The RfD (mg/kg day) values of Cd, Ni, Cu, Pb, and Zn used in the present study for ingestion, inhalation, and dermal contact, are presented in Table 3. If the ADD is less than the RfD value (HQ ≤ 1), it is viewed as that there will be no adverse health effects, while if the ADD surpasses the RfD value (HQ > 1), there will likely be harmful health effects [70,72].

The NCR is assessed by HI, which is the summation of the HQs in the three exposure pathways [76,77,78]. A HI of <1.0 was expected to show that there was no significant risk of non-carcinogenic effects. A HI of >1.0 was expected to show that there was a possible occurrence of non-carcinogenic effects. There is a probability of non-carcinogenic effects having a positive connection with the increment of the HI value [22]. The HI was calculated according to Equation (9).

(9) $$\mathrm{HI}=\sum {\mathrm{HQ}}_{i}=\sum (\frac{{\mathrm{ADD}}_{i}}{{\mathrm{RfD}}_{i}})$$

The CR is evaluated by the total cancer risk values of PTMs, which is the result of the ADD and its corresponding slope factor (SF). The CR is the probability that an individual will develop cancer per unit exposure level of mg/kg day because of exposure to carcinogenic hazards over the individual’s lifetime [22]. In this study, only values of carcinogenicity slope factor (SF) inhalation for Cd (6.30) and Ni (0.84) were available [21,76]. Thus, only CR inhalation (CR_inh_) for Cd and Ni was calculated. The CR_inh_ was calculated using Equation (10).

(10) $${\mathrm{CR}}_{\mathrm{inh}}=\sum {\mathrm{ADD}}_{\mathrm{inh}}\times {\;\mathrm{SF}}_{\mathrm{inh}}$$

If CR < 1 × 10^−6^, the CR to health from the soil is negligible, and a CR > 1 × 10^−4^ is probably in high risk of causing cancer in humans. A CR value within a range from 1 × 10^−6^ to 1 × 10^−4^ shows an acceptable or tolerable risk to human health [24,25].

### Data Analysis

All statistical calculations were done by using the STATISTICA (Version 10; StatSoft. Inc., Tulsa, OK, USA, 1984–2011). Comparisons between sites and different geochemical fractions of topsoils were calculated using the One-way ANOVA analysis.

## 4. Results and Discussion

### 4.1. Potentially Toxic Metals in Topsoils

The concentrations of PTMs in topsoil sampled from Peninsular Malaysia are presented in Table 4. The metal concentrations (mg/kg dry weight) were 0.24–12.4 for Cd (mean: 1.94), 4.66–2363 for Cu (mean: 228), 2576–116,344 for Fe (mean: 32618), 2.38–75.7 for Ni (mean: 16.0), 7.22–969 for Pb (mean: 115), and 11.0–3820 for Zn (mean: 512).

L-3 was found to have the highest Cd (12.4 mg/kg) and Zn (3820 mg/kg) concentrations among the sampling sites, whereas the highest Cu (2363 mg/kg) and Pb (969 mg/kg) concentrations (*p* < 0.05) were detected in RH-3. L-3 contained the highest in Ni concentration (75.7 mg/kg).

Their comparisons with pre-industrial reference levels and UCC levels are also presented in Table 4. The overall mean Cd of the six different land uses were all above those of the pre-industrial reference levels [65]: UCC limits by Taylor and McLennan [79], Rudnick and Gao [80], and Wedepohl [48,81]. However, all the mean Ni concentrations of all land uses were below those of the UCC limits by Taylor and McLennan [79], Rudnick and Gao [80] and Wedepohl [48], except those by Wedepohl [81] for mean Ni values of the landfill and rubbish heap (exceeded 19 mg/kg dry weight). Except for the residential and plantation areas, the overall mean values of Cu and Zn of all the other land uses were higher than those by all the reference values. The sites from industrial, landfill, and rubbish heap exceeded the reference values of Pb.

Lastly, the mean Fe levels were in the following order: mining (64,606) > industrial (39,315) > plantation (38,891) > residential (30,738) > rubbish heap (26,039) > landfill (24,186). The abandoned mining area exceeded the Fe UCC values by Wedepohl [48,81], while the industrial and plantation areas exceeded the Fe UCC values by Wedepohl [81].

### 4.2. Assessment of Potentially Toxic Metals Pollution

#### 4.2.1. Geoaccumulation Index

The results from the tabulation of I_geo_ values of all sampling sites are presented in Table 5. For Cd, all I_geo_ values of all sites ranged from 0.68 “unpolluted” to 6.37 “very strongly polluted”. For Cu, all I_geo_ values of all sites ranged from −3.01 “practically unpolluted” to 5.98 “very strongly polluted”. For Ni, all Igeo values were below 1.0 (−5.14 to −0.15) (“practically unpolluted”). For Pb, all I_geo_ values of all sites ranged from −1.64 “practically unpolluted” to 5.43 “very strongly polluted”. For Zn, all I_geo_ values of all sites ranged from −3.15 “practically unpolluted” to 5.29 “very strongly polluted”. In particular, site I-1 recorded Cd (4.73), Pb (3.54), and Zn (4.60), site L-3 recorded Cd (6.37), Cu (5.55), Pb (4.46), and Zn (5.29), and site RH-3 recorded Cd (5.65), Cu (5.98), Pb (5.43), and Zn (4.93). This clearly shows that sites I-1, L-3 and RH-3 are categorized as “strongly polluted” (3–4), to “very strongly polluted” (>5).

#### 4.2.2. Pollution Load Index

The results from the tabulation of the PLI values based on Cd, Cu, Ni, Pb, and Zn in all sampling sites topsoils are presented in Table 5. The PLI values ranged from 0.38–29.6 (mean: 4.34) in all the sampling sites. The mean value of 4.34 indicated that Peninsular Malaysia soils were “highly polluted” (4 < PLI ≤ 5). In particular, sampling sites categorized as “very highly polluted” (PLI > 5) were I-1, L-3, and RH-3. A sampling site categorized as “highly polluted” (4 < PLI ≤ 5) is M-1. Sampling sites categorized as “moderately to highly polluted” (3 < PLI ≤ 4), were R-6 and RH-2. Therefore, the PLI values complemented the results of I_geo_ in which topsoils sampled from I-1, L-3, and RH-3 were detected to have a higher contamination degree when compared with the other sites.

#### 4.2.3. Ecological Risk and Potentially Ecological Risk Index

Values of ER for Cd, Cu, Ni, Pb, and Zn, and PERI on topsoil collected from different land uses in Peninsular Malaysia are presented in Table 5. The values of ER based on 23 sites ranged from 72.0 “moderate potential ecological risk” to 3720 “very high ecological risk” for Cd, 0.93 “low potential ecological risk” to 473 “very high ecological risk” for Cu, 0.21 to 6.76 “low potential ecological risk” for Ni, 2.41 “low potential ecological risk” to 323 “very high ecological risk” for Pb, and 0.17 “low potential ecological risk” to 58.8 “moderate potential ecological risk” for Zn (Table 5).

Of particular concern, sites I-1, L-3, M-1, and RH-3 recorded “very high ecological risk” (PERI ≥ 600), according to Hakanson [65]. I-1 was an industrial area, L-3 was located in the vicinity of the landfill areas, and M-1 was an abandoned mining location. The rubbish heap at RH-3 was visibly observed to be contributed by municipal wastes including electronic waste from nearby locations. The above site descriptions could explain the reason of “very high ecological risk”.

### 4.3. Comparisons of PERI with Other Studies

Comparisons of PTMs concentrations, I_geo_, PLI, ER, and PERI values of topsoils between reported studies and the present finding are presented in Table 6. The comparison of I_geo_, CF, PLI, ER, and PERI values with other studies in Table 6 becomes more comparable and relevant because the cited PTMs data in the soils from literature in Table 6 were recalculated for the values of I_geo_, CF, PLI, ER, and PERI. The calculations were based on the similar background metal concentrations in the earth’s UCC proposed by Wedepohl [48], and the toxic response factor (T_R_) of the five metals according to Hakanson [65].

The overall mean values of I_geo_, based on 23 sites were Cd (2.93; “moderately polluted to strongly polluted”), Cu (−0.29; “practically unpolluted”), Ni (−2.93; “practically unpolluted”), Pb (1.31; “moderately polluted”), and Zn (0.26; “unpolluted”) (Table 6).

Out of 17 comparisons in Table 6 for Cd I_geo_, the present mean Cd I_geo_ of all sampling sites of Peninsular Malaysia was lower than those reported for Dabaoshan mine [67], Khatoon Iran [82], Kuala Terengganu [83], Recife metropolitan region [68], habitat topsoils of *Centella asiatica* from Peninsular Malaysia (Ong et al. 2016), Seri Kembangan industrial area [84], mining area in Huiza of China [85], and farmland area Xinxiang [86].

Out of 20 comparisons in Table 6 for Cu I_geo_, the present mean Cu of all sampling sites of Peninsular Malaysia was comparable to 9 comparisons and lower than the other 11 comparisons in Table 6. Out of 13 comparisons in Table 6 for Ni I_geo_, the present mean Ni of all sampling sites of Peninsular Malaysia was comparable to 8 comparisons and lower than those of the other 5 comparisons in Table 6. This shows that Cu and Ni status in topsoils of Peninsular Malaysia with different land uses is practically unpolluted.

Out of 20 comparisons in Table 6 for Pb Igeo, the present mean Pb I_geo_ of all sampling sites of Peninsular Malaysia was higher in 13 comparisons but lower than those reported for Dabaoshan mine [67], Khatoon Iran [82], Hyderabad industrial area of India [61], habitat topsoils of *Centella asiatica* from Peninsular Malaysia [87], Seri Kembangan industrial area [84], mining area in Huize of China [85], and Bestari mine dump [17].

Out of 20 comparisons in Table 6 for Zn I_geo_, the present mean Zn Igeo of all sampling sites of Peninsular Malaysia was only higher than those in Kuala Terengganu [83], Bestari ex tin mining [17], and Peninsular Malaysia agricultural crop soils [88]. The mostly lower Zb Igeo in comparison to the other 16 comparisons indicates that the Zn status in topsoils of Peninsular Malaysia can be categorized as unpolluted. Overall, all the I_geo_ values of Cd, Cu, Ni, Pb, and Zn were dominated by industry, landfill, mining, and rubbish heap.

The PLI range based on Cd, Cu, Ni, Pb, and Zn of all sampling sites was 0.38 “unpolluted”, to 29.6 “very highly polluted” with a mean value of 4.34 “highly polluted” (Table 6). Out of 19 publications with 21 comparisons in Table 6, the mean PLI value from the present study was higher than those of 14 comparisons. The present PLI value was lower than those reported for Dabaoshan, Linxiang, and Daye of China [67], a mining site at Huize County (China) [85], a copper smelter site at Khatoon Abad (Iran) [82], a farmland at Xinxiang City (China) [86], a dump mine site at Bestari Jaya (Malaysia) [17], agricultural crop soils of Peninsular Malaysia Zarcinas et al. [88], and Seri Kembangan industrial site (Malaysia) [84]. When we investigated the land uses, the PERI values were dominated by mining (mean PERI: 892), rubbish heap (mean PERI: 1263), landfill (mean PERI: 1335), and industry (mean PERI: 1338).

The overall mean values of ER based on 23 sites were Cd (582), Cu (45), Ni (1.42), Pb (38.3), and Zn (7.88) (Table 6). The ER value of Cd was high (ER > 160) for most sampling sites (18 out of 23 sites; 78.3%) (Table 5) This is in good agreement with that reported by Qing et al. [21], which was 90% Cd ER. The outcomes showed the “high potential ecological risk” of Cd that could pose to the human body and the biological ecosystem. As indicated by other authors [1], Cd contributed significantly to the PERI of the environment. The PERI for single metal demonstrated that the severity of pollution of the five metals diminished in the accompanying succession: Cd > Cu > Pb > Zn > Ni. The present finding was comparable to the sequence based on Anshan soils, which was Cd > Cu > Pb > Ni > Zn as reported by Qing et al. [21].

The overall mean PERI value was recorded as 675 (Table 6), which is categorized as “very high ecological risk” (PERI ≥ 600), according to Hakanson [65]. Out of 19 publications with 21 comparisons in Table 6, the mean PERI value from the present was higher than those of 15 comparisons. The present PERI value was lower than those reported for Dabaoshan, Linxiang, and Daye of China [67], a dump mine site at Bestari Jaya (Malaysia) [17], a copper smelter site at Khatoon Abad (Iran) [82], a farmland at Xinxiang City, China) [86], a mining site at Huize County (China) [85], and Seri Kembangan industrial site (Malaysia) [84].

When we investigated the land uses, the values of PLI and PERI were dominated by mining, rubbish heap, landfill, and industry. In particular, the high PERI (1338) found in the industrial area in Juru (I-1), which exceeded the values in Hyderabad industrial area [61], Anshan industrial city [21], and Panzhihua industrial mining city [55].

For landfill areas, Cd, Ni, and Pb levels were generally within the ranges from Hyderabad (industrial area) [61], Xinxiang City (farmland) [86], Recife Metropolitan region of Brazil [68], and Seri Kembangan (urban area) [84] as presented in Table 6. However, L-3 had Cu (1754 mg/kg) and Zn (3820 mg/kg) level that exceeded the metal ranges in the industrial area of Hyderabad and the farmland of Xinxiang city. This suggests that the topsoil was heavily polluted with high PERI values of 252 (Cu) and 58.8 (Zn). These PERI values were higher than those reported in the industrial area of Hyderabad, and the farmland of Xinxiang city.

For the topsoils collected from the rubbish heap area, RH-3 exceeded the range of the Recife Metropolitan region for Cd (7.49 mg/kg), Cu (2363 mg/kg), Ni (57.7 mg/kg), and Pb (969 mg/kg). The levels of Cu and Zn in RH-3 also exceeded the metal ranges in the industrial areas of Hyderabad and the farmland of Xinxiang City. The PERI value of RH-3 exceeded the range of PERI from the industrial area of Hyderabad.

For the abandoned tin mining site, the PERI of M-1 exceeded the metal ranges from mining area of several studies, namely Huize county [85], Kuala Lipis [69], and Bukit Ibam [69]. However, the concentrations of Cu, Pb, and Zn were still within the metal ranges from the study on mine dumps in Bestari Jaya [17]. The PERI value in the present study was close to that reported for a mining site in Huize County [85].

### 4.4. Human Health Risk Assessment

**Figure 2:**
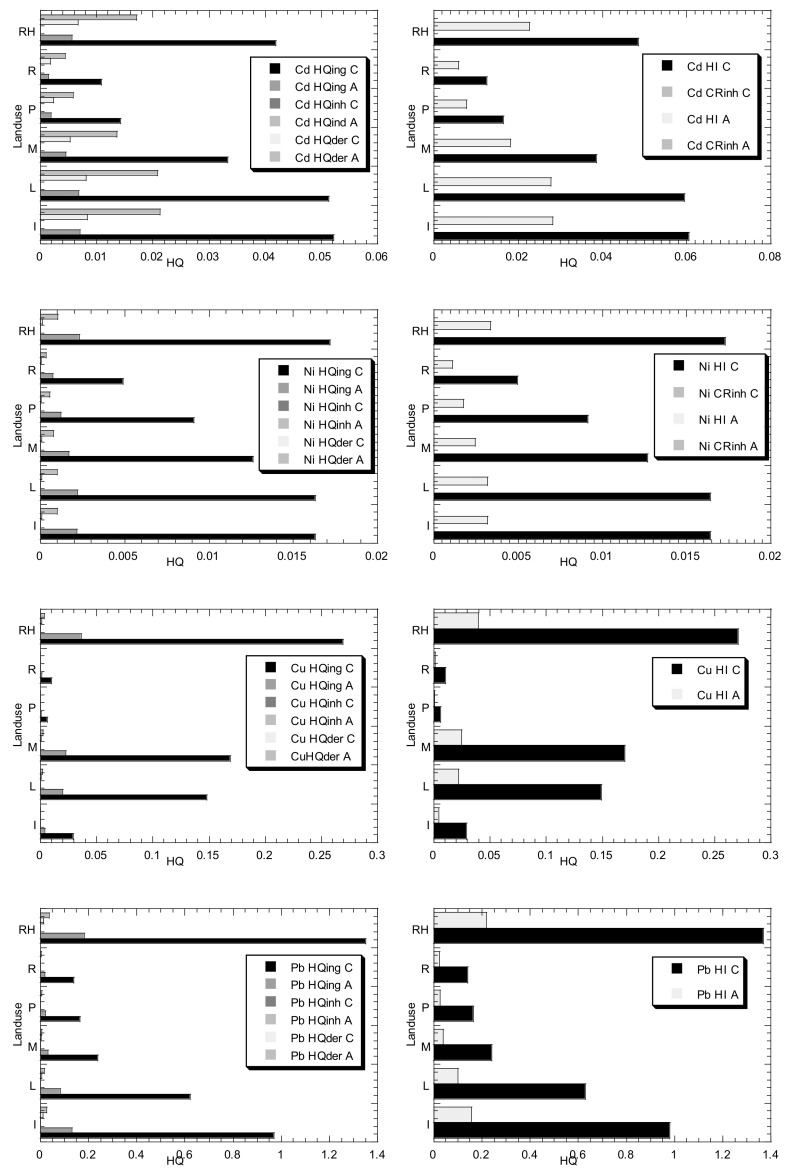

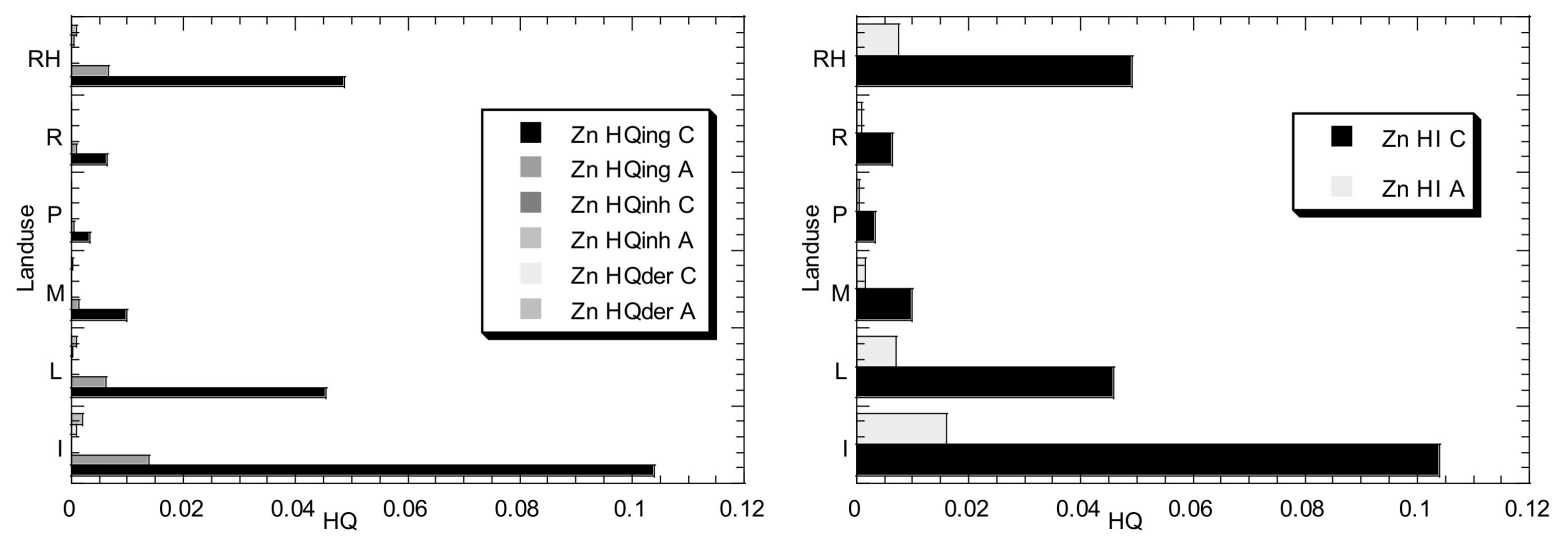
Comparisons of the values of hazard quotient (HQ), and hazard index (HI), in the three exposure routes of Cd, Ni, Cu, Pb, and Zn in children (C) and adults (A) from the present study. Note: The values of carcinogenic risk (CR_inh_) were also calculated for Cd and Ni. (Note: The RfD*_ing_* (mg/kg day) value used in the present study is 1.00 × 10^−3^ for Cd. The RfD*_inh_* (mg/kg day) value used in the present study is 1.00 × 10^−3^ for Cd. The RfD*_der_* (mg/kg day) value used in the present study is 1.00 × 10^−5^ for Cd. The SF*_inh_* (mg/kg day)^−1^ value used in the present study is 6.30 for Cd. The RfD*_ing_* (mg/kg day) value used in the present study is 2.00 × 10^−2^ for Ni. The RfD*_inh_* (mg/kg day) value used in the present study is 2.06 × 10^−2^ for Ni. The RfD*_der_* (mg/kg day) value used in the present study is 5.40 × 10^−3^ for Ni. The SF*_inh_* (mg/kg day)^−1^ value used in the present study is 8.40 × 10^−1^ for Ni. The RfD*_ing_* (mg/kg day) value used in the present study is 4.00 × 10^−2^ for Cu. The RfD*_inh_* (mg/kg day) value used in the present study is 4.02 × 10^−2^ for Cu. The RfD*_der_* (mg/kg day) value used in the present study is 1.20 × 10^−2^ for Cu. The SF*_inh_* (mg/kg day)^−1^ value is not available for Cu and therefore, carcinogenic risk inhalation (CR_inh_) for Cu is not calculated. The RfD*_ing_* (mg/kg day) value used in the present study is 3.50 × 10^−2^ for Pb. The RfD*_inh_* (mg/kg day) value used in the present study is 3.52 × 10^−3^ for Pb. The RfD*_der_* (mg/kg day) value used in the present study is 5.25 × 10^−4^ for Pb. The SF*_inh_* (mg/kg day)^−1^ value is not available for Pb and therefore, carcinogenic risk inhalation (CR_inh_) for Pb is not calculated. The RfD*_ing_* (mg/kg day) value used in the present study is 3.00 × 10^−1^ for Zn. The RfD*_inh_* (mg/kg day) value used in the present study is 3.00 × 10^−1^ for Zn. The RfD*_der_* (mg/kg day) value used in the present study is 6.00E−02for Zn. The SF*_inh_* (mg/kg day)^−1^ value is not available for Zn and therefore, carcinogenic risk inhalation (CR_inh_) for Zn is not calculated.

The HHRA results due to PTMs exposures in the topsoils of different land uses in Peninsular Malaysia are shown in Figure 2. For children Cd (Figure 2), based on the mean values of the six different land uses, the HQ_ing_ values ranged from 1.09 × 10^−2^ to 5.22 × 10^−2^, the HQ_der_ values ranged from 1.74 × 10^−3^ to 8.35 × 10^−3^, while HQ_inh_ values ranged from 2.98 × 10^−7^ to 1.43 × 10^−6^. For adult Cd (Figure 2), based on the mean values of the six different land uses, the HQ_ing_ values ranged from 1.46 × 10^−3^ to 7.00 × 10^−3^, the HQ_der_ values ranged from 4.45 × 10^−3^ to 2.13 × 10^−2^, while HQ_inh_ values ranged from 1.34 × 10^−7^ to 6.43 × 10^−7^. The values of HQ_ing_ and HQ_der_ were higher in children than those in adults, while the values of HQ_inh_ were higher in adults than those in children. All the three Cd pathways followed Industrial > landfill > rubbish heap > mining > plantation > residential. For the children Cd CR_inh_ values, the six land uses ranged from 5.42 × 10^−10^ to 8.99 × 10^−9^ while those for adults ranged from 2.44 × 10^−10^ to 4.05 × 10^−9^. The CR_inh_ values of Cd for children and adults were lower than 10^−6^, indicating that the CR of Cd in the topsoils collected from the six land uses from Peninsular Malaysia could be neglected. The present Cd CR_inh_ values were comparable to Qing et al. [21]’s findings, who reported that the Cd CR_inh_ values in the urban soils of Anshan were 1.94 × 10^−9^ and 8.75 × 10^−10^ for children and adults, respectively. Zhao et al. [30] reported that the spatial pattern of the HQs in the soils near Dabaoshan Mine indicated that Cd was the most important PTM contributing to the HHR.

For children Ni (Figure 2), based on the mean values of the six different land uses, the HQ_ing_ values ranged from 4.92 × 10^−3^ to 1.72 × 10^−2^, the HQ_der_ values ranged from 4.08 × 10^−5^ to 1.02 × 10^−4^, while HQ_inh_ values ranged from 1.38 × 10^−7^ to 4.58 × 10^−7^. For adult Ni (Figure 2), based on the mean values of the six different land uses, the HQ_ing_ values ranged from 7.55 × 10^−4^ to 2.31 × 10^−3^, the HQ_der_ values ranged from 3.40 × 10^−4^ to 1.04 × 10^−3^, while HQ_inh_ values ranged from 2.69 × 10^−7^ to 8.25 × 10^−7^. The values of HQ_ing_ and HQ_der_ were higher in children than those in adults, while the values of HQ_inh_ were higher in adults than those in children. All the three Ni pathways followed rubbish heap > landfill > Industrial > mining > plantation > residential. For the children Ni CR_inh_ values, the six land uses ranged from 7.17 × 10^−10^ to 1.74 × 10^−8^ while those for adults ranged from 1.29 × 10^−9^ to 3.13 × 10^−8^. The CR_inh_ values of Ni for children and adults were lower than 10^−6^, indicating that the CR_inh_ of Ni in the topsoils collected from the six land uses from Peninsular Malaysia could be neglected. The present Ni CR_inh_ values were comparable to Qing et al. [21]’s findings too, in which the Ni CR_inh_ values in the urban soils of Anshan were 1.01 × 10^−8^ and 4.54 × 10^−9^ for children and adults, respectively.

For children Cu (Figure 2), based on the mean values of the six different land uses, the HQ_ing_ values ranged from 6.03 × 10^−3^ to 2.69 × 10^−1^, the HQ_inh_ values ranged from 1.64 × 10^−7^ to 7.33 × 10^−6^, while HQ_der_ values ranged from 3.21 × 10^−5^ to 1.44 × 10^−3^. For adult Cu (Figure 2), based on the mean values of the six different land uses, the HQ_ing_ values ranged from 8.08 × 10^−4^ to 3.61 × 10^−2^, the HQ_inh_ values ranged from 7.38 × 10^−8^ to 3.30 × 10^−6^, while HQ_der_ values ranged from 8.21 × 10^−5^ to 3.66 × 10^−3^. The values of HQ_ing_ and HQ_inh_ were higher in children than those in adults, while the values of HQ_der_ were higher in adults than those in children. All the three Cu pathways followed rubbish heap > mining > landfill > Industrial > residential > plantation.

For children Pb (Figure 2), based on the mean values of the six different land uses, the HQ_ing_ values ranged from 1.39 × 10^−1^ to 1.35, the HQ_inh_ values ranged from 3.82 × 10^−6^ to 3.72 × 10^−5^, while HQ_der_ values ranged from 1.50 × 10^−3^ to 1.46 × 10^−2^. For adult Pb (Figure 2), based on the mean values of the six different land uses, the HQ_ing_ values ranged from 1.86 × 10^−2^ to 1.81 × 10^−1^, the HQ_inh_ values ranged from 1.72 × 10^−6^ to 1.67 × 10^−5^, while HQ_der_ values ranged from 3.82 × 10^−3^ to 3.72 × 10^−2^. The values of HQ_ing_ and HQ_inh_ were higher in children than those in adults, while the values of HQ_der_ are higher in adults than those in children. All the three Pb pathways followed rubbish heap > Industrial > landfill > mining > plantation > residential.

For children Zn (Figure 2), based on the mean values of the six different land uses, the HQ_ing_ values ranged from 3.23 × 10^−3^ to 1.04 × 10^−1^, the HQ_inh_ values ranged from 8.84 × 10^−8^ to 2.83 × 10^−6^, while HQ_der_ values ranged from 2.58 × 10^−5^ to 8.28 × 10^−4^. For adult Zn (Figure 2), based on the mean values of the six different land uses, the HQ_ing_ values ranged from 4.33 × 10^−4^ to 1.39 × 10^−2^, the HQ_inh_ values ranged from 3.98 × 10^−8^ to 1.27 × 10^−6^, while HQ_der_ values ranged from 6.60 × 10^−5^ to 2.11 × 10^−3^. The values of HQ_ing_ and HQ_inh_ were higher in children than those in adults, while the values of HQ_der_ were higher in adults than those in children. All the three Zn pathways followed Industrial > rubbish heap > landfill > mining > residential > plantation.

It was shown that the three different exposure pathways of Cd, Cu, Ni, Pb, and Zn for children and adults diminished in the following order: ingestion > dermal contact > inhalation. The contribution of HQ*_ing_* to HI (total risk of non-carcinogenic) was the highest; Zn (99.2% and 86.8% for children and adults, respectively), Pb (98.9% and 83.0% for children and adults, respectively), Cu (99.5% and 90.8% for children and adults, respectively), Ni (99.4% and 68.9% for children and adults, respectively), and Cd (86.2% for children). However, the Cd contribution of HQ*_ing_* to HI was only 24.7% for adults. The highest Cd contribution (75.3%) to HI for adults was found in HQ_der_.

These percentages were comparable to those reported in Anshan by Qing et al. [21], namely an average of 96.5% for children and 72.5% for adults based on Cr, Cd, Cu, Pb, Ni and Zn. This emphatically recommended that ingestion was the fundamental exposure pathway to undermine human health. This outcome was likewise predictable with those revealed from India [20], and street dust in Beijing [23]. Gu et al. [25] likewise found that for NCR, the ingestion of soil particles happened to be the major pathway through which health risks were caused to occupants of Guangzhou. Comparable outcomes had been reported in other urban areas [76,78]. Gu et al. [25] reported that the relative contribution of ingestion to the HI values ranged from 77.9 to 99.1% and 71.0 to 98.7%, for children and adults, respectively.

The HI values for all the five metals were < 1.0 (Figure 2), indicating that there was no NCR for children and adults. By comparing the HI values for children and adults, it could be summarized that children had higher chances of NCR from PTMs in the rubbish heap, landfill and industrial sites than those in adults. The higher NCR in children than adults was generally due to their pica behavior, and hand or finger sucking [23,30].

Due to the absence of the carcinogenic slope factors for Pb, Cu, and Zn, only the CR_inh_ values for Cd and Ni were estimated. Similar to HI values, CR_inh_ values of Cd and Ni for children were also higher compared to those of adults. The values for both non-carcinogenic (Cd, Cu, Ni, Pb, and Zn) and CR_inh_ (Cd and Ni) obtained for adults in this study were all within the acceptable range, indicating no serious adverse impacts on children and adults’ health. The CR values of Cd and Ni from exposure to the urban park soils from Guangzhou decreased in the order Ni > Cd (Gu et al. 2016), which is in good agreement with the present finding.

Of public concern, the HI for Pb children at the landfill (L-3) and rubbish heap (RH-3) sites exceeded 1.0, indicating non-carcinogenic risk for children. L-3 was a landfill site that could pose an unhealthy non-carcinogenic risk (HI > 1 for Pb). RH-3 was a rubbish heap site with domestic rubbish. This area was found nearby to residential and shop areas, a higher level of visiting pedestrian was expected. This site posed an unhealthy non-carcinogenic risk of Pb (HI > 1) to children. It is not recommended to let children play in that site for their safety concerns.

The child group was exposed to a greater risk of the adverse health effects from the influence of the contaminants. The results estimated that child group risk was mostly caused by dermal absorption of the contaminants. The increment of the non-carcinogenic health risk was directly related to the exposed skin areas on the human body. In this situation, this landfill was not situated in a crowded area, hence there was no concern to the general public. However, children generally have higher health risk exposure to the surrounding pollutant due to their behavior and physiology. They have a higher hand to mouth activities, higher respiration rates, and increased gastrointestinal absorption of some substances [89]. Adedeji et al. [28] investigated the soils around the Gateway Trailer Park (Nigeria) and reported very high PERI, while total HI and CR indicated that children had the highest health risk. Ning et al. [29] also reported that the health risk to children exceeded that of adults, based on soil samples, collected from a historic TlHg mining area, located in southwest Guizhou (China).

The present findings indicated that the land uses of soils in Peninsular Malaysia had affected the accumulation of heavy metals in soils, which could endanger ecological safety and human health [46,51]. This study increased our understanding of PTM pollution in soil that could potentially harm the environment and human health in Peninsular Malaysia [52]. Moreover, different indices (EF, PLI, and ER) played a critical role in the integrated assessment of soil PTM pollution, ecological and health risk assessment, and provided an empirical basis for the sustainable future planning and comprehensive adaptive management practices [54] for Peninsular Malaysia. Thus, the land uses involving industrial, landfill, mining, and rubbish heap should be regarded as a priority to reduce health risk in Peninsular Malaysia [3]. This study provided more comprehensive information for better soil management and pollution control in Peninsular Malaysia.

## 5. Conclusions

The concentrations, pollution, ecological risks and HHRA of Cd, Pb, Ni, Cu, and Zn in the topsoils of six land uses in Peninsular Malaysia were investigated in the present study. For the PTMs pollution assessment, I-1 was found to have “extremely high enrichment” in Cd and Zn. The EF values of Cd, Cu, and Zn in L-3 were categorized as “extremely high enrichment”. The EF values of Cd, Cu, Pb, and Zn in RH-3 were categorized as “extremely high enrichment”. In general, topsoils sampled from I-1 (industrial), L-3 (landfill), and RH-3 (rubbish heap) were detected to have higher values of PLI and I_geo_ compared with the other sites.

For the ecological risk assessment, the PERI for single metal indicated that the severity of pollution of the five metals decreased in the following sequence: Cd > Cu > Pb > Zn > Ni. The overall mean values of PERI based on land uses were in the following sequence: industrial > landfill > rubbish heap > mining > plantation > residential areas. The first four land uses were categorized as “very high ecological risk”. This was well indicated at sites I-1, L-3, and RH-3.

For HHRA, the land uses of the industrial, landfill, and rubbish heap areas were found to have higher HQ values of the three pathways, when compared to mining, plantation, and residential areas. It was indicated that the three different exposure pathways of Cd, Cu, Ni, Pb, and Zn for children and adults decreased in the following order: ingestion > dermal contact > inhalation.

In general, the HI values for all the metals in all sites of the six different land uses were lower than 1, indicating that there was no non-carcinogenic risk for children and adults. However, of public concern was that the HI for Pb children at the landfill (L-3) and rubbish heap (RH-3) sites exceeded 1.0, indicating non-carcinogenic risk for children. The CR_inh_ values of Cd and Ni for children and adults were lower than 10^−6^, indicating that the CR_inh_ of Cd and Ni in the topsoils collected from the six land uses could be neglected. Therefore, this reflected the fact that there were no serious adverse impacts on children’s and adults’ health from the six land uses from Peninsular Malaysia. However, to protect human health and well-being, continual HHRA of PTMs at different land uses in Malaysia is warranted. This should become the major agenda in nation building in line with the sustainable development goals.

## Figures and Tables

**Table 1:** Sampling information for topsoils collected from different land uses in Peninsular Malaysia.

Land Uses		Sampling Site	N	E	Date	Weather Condition	Time	Distance from the Road (m)
Industrial	I-1	Juru	5°20′56.00″	100°24′45.10″	2 August 2011	Sunny	5.00 p.m.	6.00
Landfill	L-1	Matang	4°49′16″	100°40′44″	27 June 2011	Cloudy	2.00 p.m.	5.00
	L-2	Sepang	2°44′57″	101°37′59″	2 July 2011	Sunny	11.25 a.m.	NA
	L-3	Sg. Kembung	2°53′8.30″	101°49′20.80″	2 July 2011	Sunny	3.00 p.m.	NA
	L-4	Tanjung Langsat Landfill, Johor	1°28′12.80″	103°59′33.10″	10 July 2011	Sunny	9.00 a.m.	0.50
Mining (abandoned)	M-1	Sg. Lembing	3°54′29.40″	103°1′50.00″	22 July 2011	Sunny	1.30 p.m.	13.0
Plantation	P-1	Kg. Ayer Hitam	4°1′33.70″	101°12′9.20″	26 June 2011	Sunny	2.30 p.m.	11.0
	P-2	Perah, Kuala Lipis	4°29′10.30″	101°57′24.60″	15 July 2011	Cloudy	1.45 p.m.	13.0
	P-3	Alor Setar (Paddy)	6°6′33.40″	100°20′10.00″	3 August 2011	Sunny	9.30 a.m.	25.0
	P-4	Pendang (Paddy)	5°59′4.80″	100°27′10.80″	3 August 2011	Drizzle	12.10 p.m.	30.0
	P-5	Tg. Gemok	2°40′4.90″	103°35′41.50″	17 November 2011	Sunny	10.30 a.m.	5.20
Residential	R-1	Kg. Bkt. Chandang	3°27′55.60″	101°38′24.40″	8 June 2011	Sunny	11.00 a.m.	6.00
	R-2	Kg. Bkt. Rasa	3°30′16.80″	101°38′0.80″	21 June 2011	Sunny	11.00 a.m.	3.50
	R-3	Ijok	3°19′38.00″	101°25′8.00″	21 June 2011	Sunny	3.30 p.m.	5.00
	R-4	Tanjung Piai	1°16′55.40″	103°30′34.70″	9 July 2011	Sunny	4.00 p.m.	3.20
	R-5	Kota Bharu	6°7′57.00″	102°14′8.20″	16 July 2011	Cloudy	8.30 a.m.	80.0
	R-6	Kuantan	3°47′31″	103°17′53″	22 July 2011	Sunny	4.00 p.m.	4.00
	R-7	Chukai/Kemaman	4°14′19.00″	103°25′19.30″	23 July 2011	Cloudy	9.15 a.m.	15.0
	R-8	Cheneh	4°8′29.70″	103°14′2.20″	23 July 2011	Cloudy	12.00 p.m.	50.0
	R-9	Pagoh	2°10′59.00″	102°44′46.00″	17 January 2012	Cloudy	11.00 a.m.	7.00
Rubbish heap	RH-1	Kuala Krai	5°33′45.40″	102°12′2.90″	15 July 2011	Cloudy	1.45 p.m.	1.00
	RH-2	Nibong Tebal	5°9′2.80″	100°27′45.90″	2 August 2011	Sunny	3.00 p.m.	NA
	RH-3	Kuala Terengganu	5°20′7.70″	103°8′12.20″	16 November 2011	Drizzle	9.00 a.m.	NA

Note: NA = Not available.

**Table 2:** Heavy metals analysis recovery percentages of the certified reference materials (CRM).

CRM	Cd	Cu	Fe	Ni	Pb	Zn
NSC DC73319 Soil China	111%	85.0%	NA	NA	99.8%	99.7%
MESS-3 NRC	NA	93.1%	NA	102%	116%	82.8%
TH-1 Sediment Canada	102%	92.9%	95.6%	112%	100%	110%
SRM 1547	NA	NA	106%	NA	NA	115%
IAEA Soil-5	156%	91.3%	NA	103%	116%	94.8%

NA—data not available.

**Table 3:** Definition, exposure factors and reference values used to estimate the intake values and health risks of potentially toxic metals in topsoils collected from Peninsular Malaysia.

Factor	Definition	Unit	Values		References
			Children	Adults	
IngR	Ingestion rate of soil	mg/day	200	100	[70]
InhR	Inhalation rate of soil	m^3^/day	7.63	12.8	[22]
BW	Bodyweight of the exposed individual	kg	15	55.9	[74]
EF	Exposure frequency	days/year	350	350	[74]
ED	Exposure duration	years	6	24	[70]
AT	Average time	days	365 × ED	365 × ED	[72]
PEF	Particle emission factor	m^3^/kg	1.36 × 10^9^	1.36 × 10^9^	[70]
SA	Exposed skin surface area	cm^2^	1600	4350	[74]
AF	Skin adherence factor	mg/cm day	0.2	0.7	[75]
ABF	Dermal absorption factor	unitless	1.00 × 10^−3^	1.00 × 10^−3^	[20]
Cd RfD	Reference dose for ingestion	mg/kg day	1.00 × 10^−3^	1.00 × 10^−3^	[21]
Cd RfD	Reference dose for inhalation	mg/kg day	1.00 × 10^−3^	1.00 × 10^−3^	[21]
Cd RfD	Reference dose for dermal contact	mg/kg day	1.00 × 10^−5^	1.00 × 10^−5^	[21]
Ni RfD	Reference dose for ingestion	mg/kg day	2.00 × 10^−2^	2.00 × 10^−2^	[21]
Ni RfD	Reference dose for inhalation	mg/kg day	2.06 × 10^−2^	2.06 × 10^−2^	[21]
Ni RfD	Reference dose for dermal contact	mg/kg day	5.40 × 10^−3^	5.40 × 10^−3^	[21]
Cu RfD	Reference dose for ingestion	mg/kg day	4.00 × 10^−2^	4.00 × 10^−2^	[21]
Cu RfD	Reference dose for inhalation	mg/kg day	4.02 × 10^−2^	4.02 × 10^−2^	[21]
Cu RfD	Reference dose for dermal contact	mg/kg day	1.20 × 10^−2^	1.20 × 10^−2^	[21]
Pb RfD	Reference dose for ingestion	mg/kg day	3.50 × 10^−3^	3.50 × 10^−3^	[21]
Pb RfD	Reference dose for inhalation	mg/kg day	3.52 × 10^−3^	3.52 × 10^−3^	[21]
Pb RfD	Reference dose for dermal contact	mg/kg day	5.25 × 10^−4^	5.25 × 10^−4^	[21]
Zn RfD	Reference dose for ingestion	mg/kg day	3.00 × 10^−1^	3.00 × 10^−1^	[21]
Zn RfD	Reference dose for inhalation	mg/kg day	3.00 × 10^−1^	3.00 × 10^−1^	[21]
Zn RfD	Reference dose for dermal contact	mg/kg day	6.00 × 10^−2^	6.00 × 10^−2^	[21]

**Table 4:** The mean of potentially toxic metal concentrations (mg/kg dry weight) in topsoils sampled in Peninsular Malaysia and their comparisons with pre-industrial reference levels and upper continental crust (UCC) levels.

Land Uses	Sites	Cd	Cu	Fe	Ni	Pb	Zn
Industrial	I-1	3.98 ^a^	88.3 ^a^	39,315 ^bcd^	24.8 ^a^	262 ^b^	2369 ^b^
Landfill	L-1	1.11 ^a^	8.42 ^a^	22,316 ^abc^	8.00 ^a^	90.3 ^a^	39.9 ^a^
	L-2	0.32 ^a^	7.76 ^a^	8941 ^ab^	3.36 ^a^	23.1 ^a^	11.1 ^a^
	L-3	12.4 ^b^	1754 ^ab^	31,599 ^bcd^	75.7 ^b^	495 ^b^	3820 ^c^
	L-4	1.78 ^a^	33.1 ^a^	33,886 ^cd^	12.4 ^a^	63.5 ^a^	294 ^a^
Mining	M-1	2.54 ^a^	517 ^a^	64,606 ^e^	19.3 ^a^	64.6 ^a^	225 ^a^
Plantation	P-1	0.81 ^a^	7.33 ^a^	8548 ^ab^	9.41 ^a^	36.2 ^a^	29.3 ^a^
	P-2	1.74 ^a^	27.5 ^a^	79,058 ^f^	16.5 ^a^	48.7 ^a^	84.9 ^a^
	P-3	1.12 ^a^	24.3 ^a^	37,595 ^cd^	19.5 ^a^	47.5 ^a^	99.4 ^a^
	P-4	1.10 ^a^	20.96 ^a^	39,188 ^cd^	13.25	45.17 ^a^	55.17 ^a^
	P-5	0.69 ^a^	11.84 ^a^	30,064 ^bcd^	10.66 ^a^	42.40 ^a^	100.81 ^a^
Residential	R-1	1.47 ^a^	156 ^a^	116,344 ^g^	11.2 ^a^	44.6 ^a^	262 ^a^
	R-2	0.24 ^a^	6.62 ^a^	17,421 ^abc^	3.44 ^a^	17.1 ^a^	11.0 ^a^
	R-3	0.78 ^a^	4.66 ^a^	10,273 ^ab^	9.19 ^a^	32.5 ^a^	19.2 ^a^
	R-4	1.06 ^a^	10.7 ^a^	27,434 ^bcd^	10.3 ^a^	34.3 ^a^	41.7 ^a^
	R-5	1.30 ^a^	28.8 ^a^	27,741 ^bcd^	16.9 ^a^	49.7 ^a^	357 ^a^
	R-6	1.41 ^a^	50.4 ^a^	37,283 ^cd^	14.2 ^a^	84.2 ^a^	505 ^a^
	R-7	0.24 ^a^	5.49 ^a^	2576 ^a^	2.39 ^a^	7.22	45.2 ^a^
	R-8	0.48 ^a^	9.19 ^a^	15,236 ^abc^	2.38 ^a^	20.2 ^a^	15.1 ^a^
	R-9	0.50 ^a^	9.55 ^a^	22,674 ^abc^	7.25 ^a^	47.25 ^a^	50.31 ^a^
Rubbish heap	RH-1	0.87 ^a^	9.81	17,429 ^abc^	6.20 ^a^	38.8 ^a^	75.5 ^a^
	RH-2	1.22	90.06	23,590	15	87.4	285
	RH-3	7.49 ^a^	2363.37 ^b^	37,099	57.71 ^ab^	969.22^c^	2981.23 ^bc^
Reference values		Cd	Cu	Ni	Pb	Zn	Fe
UCC [48]		0.100	25.0	56.0	15.0	65.0	43,000
Pre-industrial reference level [65]		1.00	50.0	NA	70.0	175	NA
UCC [79]		0.098	25.0	44.0	17.0	71.0	NA
UCC [80]		0.090	28.0	47.0	17.0	67.0	NA
UCC [81]		0.102	14.3	19.0	17.0	52.0	30,900

Note: Metal concentrations of different sampling sites sharing a common letter are not significantly different (*p* < 0.05) via One-Way ANOVA. NA = not available.

**Table 5:** Values of geoaccumulation index (I_geo_), contamination factor (CF), pollution load index (PLI), ecological risk (ER) for Cd, Cu, Ni, Pb, and Zn, and potentially ecological risk index (PERI) of topsoils collected from different land uses in Peninsular Malaysia (unitless).

Land Uses	Sites	Cd I_geo_	Cu I_geo_	Ni I_geo_	Pb I_geo_	Zn I_geo_	Cd CF	Cu CF	Ni CF	Pb CF	Zn CF	PLI	Cd ER	Cu ER	Ni ER	Pb ER	Zn ER	PERI
Industrial	I-1	4.73	1.24	−1.76	3.54	4.60	39.8	3.53	0.44	17.5	36.4	8.31	1194	17.7	2.21	87.3	36.4	1338
Landfill	L-1	2.89	−2.15	−3.39	2.00	−1.29	11.1	0.34	0.14	6.02	0.61	1.15	333	1.68	0.71	30.1	0.61	366
	L-2	1.09	−2.27	−4.64	0.04	−3.13	3.20	0.31	0.06	1.54	0.17	0.44	96	1.55	0.30	7.70	0.17	106
	L-3	6.37	5.55	−0.15	4.46	5.29	124	70.16	1.35	33.0	58.8	29.6	3720	351	6.76	165	58.8	4301
	L-4	3.57	−0.18	−2.76	1.50	1.59	17.8	1.32	0.22	4.23	4.52	2.51	534	6.62	1.11	21.2	4.52	567
	Mean	3.48	0.23	−2.74	2.00	0.62	39.0	18.0	0.44	11.2	16.0	8.43	1171	90.2	2.22	56.0	16.0	1335
Mining	M-1	4.08	3.79	−2.12	1.52	1.21	25.4	20.7	0.34	4.31	3.46	4.86	762	103	1.72	21.5	3.46	892
Plantation	P-1	2.43	−2.36	−3.16	0.69	−1.73	8.10	0.29	0.17	2.41	0.45	0.85	243	1.47	0.84	12.1	0.45	258
	P-2	3.54	−0.45	−2.35	1.11	−0.20	17.4	1.10	0.29	3.25	1.31	1.89	522	5.50	1.47	16.2	1.31	547
	P-3	2.90	−0.63	−2.11	1.08	0.03	11.2	0.97	0.35	3.17	1.53	1.79	336	4.86	1.74	15.8	1.53	360
	P-4	2.87	−0.84	−2.66	1.01	−0.82	11.0	0.84	0.24	3.01	0.85	1.41	330	4.19	1.18	15.1	0.85	351
	P-5	2.20	−1.66	−2.98	0.91	0.05	6.90	0.47	0.19	2.83	1.55	1.22	207	2.37	0.95	14.1	1.55	226
	Mean	2.79	−1.19	−2.65	0.96	−0.54	10.9	0.74	0.25	2.93	1.14	1.43	328	3.68	1.24	14.7	1.14	348
Residential	R-1	3.29	2.06	−2.91	0.99	1.43	14.7	6.24	0.20	2.97	4.03	2.94	441	31.20	1.00	14.9	4.03	492
	R-2	0.68	−2.50	−4.61	−0.40	−3.15	2.40	0.26	0.06	1.14	0.17	0.38	72	1.32	0.31	5.70	0.17	80
	R-3	2.38	−3.01	−3.19	0.53	−2.34	7.80	0.19	0.16	2.17	0.30	0.69	234	0.93	0.82	10.8	0.30	247
	R-4	2.82	−1.81	−3.03	0.61	−1.23	10.6	0.43	0.18	2.29	0.64	1.04	318	2.14	0.92	11.4	0.64	333
	R-5	3.12	−0.38	−2.31	1.14	1.87	13.0	1.15	0.30	3.31	5.49	2.42	390	5.76	1.51	16.6	5.49	419
	R-6	3.23	0.43	−2.56	1.90	2.37	14.1	2.02	0.25	5.61	7.77	3.16	423	10.1	1.27	28.1	7.77	470
	R-7	0.68	−2.77	−5.14	−1.64	−1.11	2.40	0.22	0.04	0.48	0.70	0.38	72	1.10	0.21	2.41	0.70	76
	R-8	1.68	−2.03	−5.14	−0.16	−2.69	4.80	0.37	0.04	1.35	0.23	0.47	144	1.84	0.21	6.73	0.23	153
	R-9	1.74	−1.97	−3.53	1.07	−0.95	5.00	0.38	0.13	3.15	0.77	0.90	150	1.91	0.65	15.8	0.77	169
	Mean	2.18	−1.33	−3.60	0.45	−0.64	8.31	1.25	0.15	2.50	2.23	1.37	249	6.25	0.77	12.48	2.23	271
Rubbish heap	RH-1	2.54	−1.93	−3.76	0.79	−0.37	8.70	0.39	0.11	2.59	1.16	1.03	261	1.96	0.55	12.9	1.16	278
	RH-2	3.02	1.26	−2.49	1.96	1.55	12.2	3.60	0.27	5.83	4.38	3.13	366	18.0	1.34	29.1	4.38	419
	RH-3	5.64	5.98	−0.54	5.43	4.93	74.9	94.5	1.03	64.6	45.9	29.3	2247	473	5.15	323	45.9	3094
	Mean	3.73	1.77	−2.26	2.72	2.04	31.9	32.8	0.47	24.3	17.1	11.2	958	164	2.35	122	17.1	1263

**Table 6:** Comparisons of values (minimum- maximum (mean)) of potential toxic metals concentrations (mg/kg dry weight), geoaccumulation index (I_geo_), contamination factor (CF), pollution load index (PLI), and ecological risk (ER) values of topsoils between reported studies and the present findings.

No.	Location (Land Use; Sampling Year; Mesh Size)	Metal	Concentrations	I_geo_	CF	PLI	ER	PERI (Mean)	References
1	Peninsular Malaysia (agricultural crops; unspecified; 2 mm)	Cd	0.01–2.02 (1.12)	−3.91–3.75 (2.90)	0.10–20.2 (11.2)	0.03–4.33 (1.13)	3.00–606 (336)	350	[88]
		Cu	0.37–114 (16.40)	−6.66–1.60 (−1.19)	0.01–4.56 (0.66)		0.07–22.80 (3.28)		
		Ni	0.40–73.5 (13.70)	−7.71–−0.19 (−1.45)	0.01–1.31 (0.24)		0.04–6.56 (1.22)		
		Pb	0.84–90 (26.4)	−2.96–2.61 (−0.51)	0.06–6.00 (1.76)		0.28–30.00 (8.8)		
		Zn	2.90–137 (38.0)	−5.07–0.49 (0.02)	0.04–2.11 (0.58)		0.04–2.11 (0.58)		
2	Bestari Jaya, Malaysia (reclaimed ex-tin mining area; 2010; 2 mm)	Cu	11.0–47.0 (29.0)	−1.77–0.33 (−0.37)	0.44–1.88 (1.16)	0.47–2.30 (1.39)	2.20–9.40 (6)	24.0	[17]
		Pb	13.0–89 (51.00)	−0.32–1.66 (0.44)	0.87–5.93 (3.40)		4.33–29.7 (17)		
		Zn	18.0–71 (44.50)	−2.44–−0.46 (0.25)	0.28–1.09 (0.68)		0.28–1.09 (1)		
3	Bestari Jaya, Malaysia (mine dumps; 2010; 2 mm)	Cu	761–2781 (1771)	4.34–6.21 (5.56)	30.4–111 (70.8)	22.1–115 (68.8)	152–556 (354)	1075	[17]
		Pb	541–3589 (2065)	4.83–7.36 (5.78)	36.1–239 (138)		180–1196 (688)		
		Zn	638–3698 (2168)	2.71–5.25 (5.85)	9.82–56.9 (33.4)		9.82–56.9 (33)		
4	Xinxiang City, China (farmland; unspecified; unspecified)	Cd	6.74–29.4 (18.1)	5.49–7.61 (6.91)	67.4–294 (181)	7.00–35.6 (21.8)	2022–8820 (5430)	5566	[86]
		Cu	29.6–133 (81.3)	−0.34–1.83 (1.12)	1.18–5.32 (3.25)		5.92–26.6 (16.3)		
		Ni	157–2090 (1124)	0.90–4.64 (4.90)	2.80–37.3 (20.1)		14.0–187 (100)		
		Zn	696–1793 (1245)	2.84–4.20 (5.05)	10.7–27.6 (19.2)		10.71–27.6 (19.2)		
5	Huize County, China (mining, 2011; 6mm)	Cd	0.10–9.50 (4.80)	−0.58–5.98 (5.00)	1.00–95.0 (48.0)	0.84–23.4 (13.2)	30.0–2850 (1440)	14,778	[85]
		Cu	14.0–52.0 (33.0)	−1.42–0.47 (−0.18)	0.56–2.08 (1.32)		2.80–10.4 (6.60)		
		Pb	4.80–2186 (1095)	3.02–4.92 (4.87)	0.32–146 (73.0)		1.60–729 (365)		
		Zn	183–679 (431)	0.91–2.80 (3.52)	2.82–10.5 (6.63)		2.82–10.5 (6.63)		
6	Seri Kembangan, Malaysia (Industry and residential; 2013; 2 mm)	Cd	11.3–67.5 (47.5)	6.24–8.81 (8.31)	113–675 (475)	24.16–500 (291)	3390–20250 (14250)	15,140	[84]
		Pb	77.5–5547 (2669)	−4.22–−2.60 (6.15)	5.17–370 (178)		25.8–1849 (890)		
7	Anshan city, China (steel industry; 2014; 0.149 mm)	Cd	0.27–1.87 (0.86)	0.85–3.64 (2.52)	2.70–18.7 (8.60)	0.69−11.2 (2.54)	81.0–561 (258)	290	[21]
		Cu	11.0–514 (52.3)	−1.77–3.78 (0.48)	0.44–20.56 (2.09)		2.20–103 (10.5)		
		Ni	13.1–49.6 (33.5)	−2.68–−0.76 (−0.16)	0.23–0.89 (0.60)		1.17–4.43 (2.99)		
		Pb	14.6–208 (45.1)	0.76–6.72 (0.27)	0.97–13.87 (3.01)		4.87–69.33 (15.03)		
		Zn	38.1–2368 (213)	−1.36–4.60 (2.51)	0.59–36.43 (3.28)		0.59–36.43 (3.28)		
8	Peninsular Malaysia (Habitat topsoils of *Centella asiatica*; 2010; 63 µm)	Cd	1.21–3.72 (1.92)	3.01–4.63 (3.68)	12.10–37.20 (19.2)	0.87–4.70 (2.56)	363–1116 (576)	625	[87]
		Cu	22.3–147 (65.9)	−0.75–1.97 (0.81)	0.89–5.88 (2.64)		4.46–29.4 (13.2)		
		Ni	4.01–13.5 (9.25)	−4.39–−2.64 (−2.02)	0.07–0.24 (0.17)		0.36–1.21 (0.83)		
		Pb	24.7–179 (98.0)	0.21–3.40 (1.39)	1.65–11.9 (6.53)		8.23–59.7 (32.7)		
		Zn	26.1–237 (130)	−1.90–1.28 (1.79)	0.40–3.65 (2.00)		0.40–3.65 (2)		
		Fe	1.37–2.79 (2.21)	-	-		-		
9	Hyderabad, India (Industrial area; unspecified; 200-mesh size)	Cu	7.90–184 (31.9)	−2.25–2.29 (−0.23)	0.32–7.36 (1.28)	0.43–13.0 (2.08)	1.58–36.8 (6.38)	69.2	[61]
		Ni	10.2–130 (43.0)	−3.04–0.63 (0.20)	0.18–2.32 (0.77)		0.91–11.6 (3.84)		
		Pb	25.3–1830 (172)	0.08–5.29 (2.20)	1.69–122 (11.5)		8.43–610 (57.3)		
		Zn	23.8–879 (108)	−2.03–3.17 (1.53)	0.37–13.5 (1.66)		0.37–13.5 (1.66)		
10	Kuala Lipis, Pahang, Malaysia (active iron ore-mining sites; 2015; 2 mm)	Cd	0.063–0.42 (0.28)	−1.25–1.49 (0.90)	0.63–4.20 (2.80)	0.67–1.80 (1.31)	18.9–126 (84)	127	[69]
		Cu	67.5–166 (110)	0.85–2.15 (1.55)	2.70–6.64 (4.40)		13.5–33.2 (22)		
		Ni	1.45–4.36 (2.93)	−5.86–−4.27 (−3.68)	0.03–0.08 (0.05)		0.13–0.39 (0.26)		
		Pb	32–72.5 (56.0)	2.05–2.37 (0.58)	2.13–4.83 (3.73)		10.7–24.2 (18.7)		
		Zn	93–116 (105)	−0.07–0.25 (1.49)	1.43–1.78 (1.62)		1.43–1.78 (1.62)		
		Fe	6.82–12.9 (10.8)	-	-		-		
11	Bukit Ibam, Pahang, Malaysia (An abandoned mine; 2015; 2 mm)	Cd	0.03–0.06 (0.04)	−2.32–−1.32 (−1.91)	0.30–0.60 (0.40)	0.49–1.30 (0.91)	9.00–18.0 (12)	51.8	[69]
		Cu	67.5–185 (145)	0.85–2.30 (1.95)	2.70–7.40 (5.80)		13.5–37.0 (29)		
		Ni	1.34–7.96 (3.91)	−5.97–−3.40 (−3.26)	0.02–0.14 (0.07)		0.12–0.71 (0.35)		
		Pb	9.65–34.5 (23.8)	2.75–2.91 (−0.66)	0.64–2.30 (1.59)		3.22–11.5 (7.93)		
		Zn	151–169 (161)	0.63–0.79 (2.10)	2.32–2.60 (2.48)		2.32–2.60 (2.48)		
		Fe	7.59–21.1 (13.50)	-	-		-		
12	Recife, Brazil (metropolitan region; unspecified; 0.149 mm)	Cd	0.00–4.3 (1.50)	0.00–4.84 (3.32)	0.00–43.0 (15.0)	0.00–20.4 (0.99)	0.00–1290 (450)	460	[68]
		Cu	0.10–1228 (12.80)	−8.55–5.03 (−1.55)	0.00–49.1 (0.51)		0.02–246 (2.56)		
		Ni	0.10–42.5 (6.30)	−9.71–−0.98 (−2.57)	0.00–0.76 (0.11)		0.01–3.79 (0.56)		
		Pb	0.10–333 (16.50)	−7.81–8.15 (−1.18)	0.01–22.2 (1.10)		0.03–111 (5.5)		
		Zn	0.10–6400 (65.20)	−9.93–6.04 (0.80)	0.00–98.5 (1.00)		0.00–98.5 (1)		
13	Kuala Terengganu (urban; unspecified; 0.6 mm)	Cd	0.38–6.78 (1.28)	1.34–5.50 (3.09)	3.80–67.8 (12.8)	0.14–5.25 (0.92)	114–2034 (384)	395	[83]
		Cu	0.82–148 (10.9)	−5.52–1.98 (−1.78)	0.03–5.92 (0.44)		0.16–29.60 (2.18)		
		Ni	1.91–16.7 (7.02)	−5.46–−2.33 (−2.42)	0.03–0.30 (0.13)		0.17–1.49 (0.63)		
		Pb	2.54–160 (23.6)	−2.29–3.18 (−0.67)	0.17–10.67 (1.57)		0.85–53.33 (7.87)		
		Zn	4.61–204 (38.3)	−4.40–1.07 (0.03)	0.07–3.14 (0.59)		0.07–3.14 (0.59)		
		Fe	0.22–7.70 (1.57)	-	-		-		
14	Southwest Guizhou, China (thallium mine area; 2018; 200-mesh)	Cd	0.14–5.17 (1.14)	−0.10–5.11 (2.93)	1.40–51.7 (11.4)	0.82–9.92 (3.74)	42.0–1551 (342)	375	[50]
		Cu	33.6–150 (79.6)	−0.16–2.00 (1.09)	1.34–6.00 (3.18)		6.72–30.00 (15.9)		
		Pb	12.7–96.5 (44.4)	−0.28–3.81 (0.24)	0.85–6.43 (2.96)		4.23–32.2 (14.8)		
		Zn	18.5–316 (118)	−2.40–1.70 (1.65)	0.28–4.86 (1.82)		0.28–4.86 (1.82)		
15	Guangzhou-Foshan, South China (urban-agriculture; unspecified; 74 μm)	Cd	0.001–1.37 (0.20)	−7.23–3.19 (0.42)	0.01–13.70 (2.00)	0.09–17.4 (0.89)	0.30–411 (60.00)	77.3	[45]
		Cu	2.37–290 (19.3)	−3.98–2.95 (−0.96)	0.09–11.60 (0.77)		0.47–58.00 (3.86)		
		Ni	2.3–442 (12.2)	−5.19–2.40 (−1.62)	0.04–7.89 (0.22)		0.21–39.46 (1.09)		
		Pb	12.3–2447 (34.8)	−0.65–4.47 (−0.11)	0.82–163 (2.32)		4.10–816 (11.6)		
		Zn	14.3–500 (45.7)	−2.77–2.36 (0.29)	0.22–7.69 (0.70)		0.22–7.69 (0.7)		
16	Ogere, Nigeria (5 land uses; 2017; 0.15 mm and 0.50 mm)	Cd	0.2–2.4 (0.70)	0.42–4.00 (2.22)	2.00–24.0 (7.00)	1.02–3.50 (1.83)	60.00–720 (210)	223	[28]
		Cu	17.9–57.6 (29.6)	−1.07–0.62 (−0.34)	0.72–2.30 (1.18)		3.58–11.52 (5.92)		
		Pb	14.4–23.4 (19.4)	1.16–2.33 (−0.95)	0.96–1.56 (1.29)		4.80–7.80 (6.47)		
		Zn	50.4–113 (68.3)	−0.95–0.21 (0.86)	0.78–1.74 (1.05)		0.78–1.74 (1.05)		
17	Harran Plain, Turkey (agriculture; 2015; 0.50 mm)	Cu	15.0–47.0 (27.0)	−1.32–0.33 (−0.47)	0.60–1.88 (1.08)	0.59–2.47 (1.06)	3.00–9.40 (5.4)	17.9	[51]
		Ni	47.0–334 (89.0)	−0.84–1.99 (1.25)	0.84–5.96 (1.59)		4.20–29.8 (7.95)		
		Pb	5.80–16.5 (10.6)	−0.58–1.06 (−1.82)	0.39–1.10 (0.71)		1.93–5.50 (3.53)		
		Zn	40.0–197 (68.0)	−1.29–1.01 (0.86)	0.62–3.03 (1.05)		0.62–3.03 (1.05)		
		Fe	2.19–6.52 (3.71)	-	-		-		
18	Khatoon Abad, Iran (copper smelter; unspecified; 63 µm)	Cd	0.2–30.4 (6.00)	0.42–7.66 (5.32)	2.00–304 (60.0)	1.14–56.5 (15.6)	60.0–9120 (1800)	2598	[82]
		Cu	38.9–10,000 (3618)	0.05–8.06 (6.59)	1.56–400 (145)		7.78–2000 (724)		
		Ni	21.2–83.2 (58.0)	−1.99–−0.01 (0.63)	0.38–1.49 (1.04)		1.89–7.43 (5.18)		
		Pb	17.5–940 (180)	2.01–7.20 (2.26)	1.17–62.7 (12.0)		5.83–313 (60)		
		Zn	90.9–3310 (548)	−0.10–5.09 (3.87)	1.40–50.9 (8.43)		1.40–50.9 (8.43)		
19	Dabaoshan, Linxiang, and Daye of China (Farmland in mining areas; unspecified; 100-mesh)	Cd	0.82–4.12 (2.67)	2.45–4.78 (4.15)	8.20–41.2 (26.7)	2.47–21.2 (10.4)	246–1236 (800)	913	[67]
		Cu	26.6–524 (241)	−0.50–3.80 (2.68)	1.06–21.0 (9.64)		5.32–105 (48.2)		
		Pb	41.4–469 (183)	2.15–4.43 (2.29)	2.76–31.3 (12.2)		13.8–156 (61)		
		Zn	100–486 (244)	0.04–2.32 (2.70)	1.54–7.48 (3.76)		1.54–7.48 (3.76)		
20	Southern Yunnan Province, China (Agriculture; 2018; 0.149mm)	Cd	0.03–4.70 (0.74)	−2.32–4.97 (2.30)	0.30–47.0 (7.40)	0.27–12.2 (2.53)	9.00–1410 (222)	260	[64]
		Cu	6.13–144 (48.3)	−2.61–1.94 (0.37)	0.25–5.76 (1.93)		1.23–28.8 (9.66)		
		Ni	4.32–197 (50.9)	−4.28–1.23 (0.44)	0.08–3.52 (0.91)		0.39–17.6 (4.54)		
		Pb	17.0–558 (67.6)	−0.58–4.46 (0.85)	1.13–37.2 (4.51)		5.67–186 (22.5)		
		Zn	15.1–495 (114)	−2.69–2.34 (1.60)	0.23–7.62 (1.75)		0.23–7.62 (1.75)		
21	Panzhihua, China (industrial mining city; unspecified; 200-mesh)	Cd	0.01–2.37 (1.10)	−3.91–3.98 (2.87)	0.10–23.7 (11.0)	0.17–11.1 (3.76)	3.00–711 (330)	326	[55]
		Cu	4.84–121 (46.2)	−2.95–1.69 (0.30)	0.19–4.84 (1.85)		0.97–24.2 (9.24)		
		Pb	0.57–142 (39.3)	−11.14–−3.25 (0.07)	0.04–9.47 (2.62)		0.19–47.3 (13.1)		
		Zn	68.9–895 (244)	−0.50–3.20 (2.70)	1.06–13.8 (3.75)		1.06–13.8 (3.75)		
22	Overall Peninsular Malaysia (6 different land uses; 2011–2012; 63 µm)	Cd	0.24–12.4 (1.94)	0.68–6.37 (2.93)	2.40–124 (19.4)	0.38–29.6 (4.34)	72.0–3720 (582)	675	This study
		Cu	4.66–2363 (228)	−3.01–5.98 (−0.29)	0.19–94.5 (9.12)		0.93–473 (45)		
		Ni	2.38–75.7 (16.0)	−5.14–−0.15 (−2.93)	0.04–1.35 (0.29)		0.21–6.76 (1.43)		
		Pb	7.22–969 (115)	−1.64–5.43 (1.31)	0.48–64.6 (7.67)		2.41–323 (38.3)		
		Zn	11.0–3820 (512)	−3.15–5.29 (0.26)	0.17–58.8 (7.88)		0.17–58.8 (7.88)		
		Fe	0.26–11.6 (3.26)	-	-		-		
23	Peninsular Malaysia (industry; 2011–2012; 63 µm)	Cd	(3.98)	(4.73)	(39.8)	(8.31)	(1194)	1338	This study
		Cu	(88.3)	(1.24)	(3.53)		(17.7)		
		Ni	(24.8)	(−0.60)	(0.44)		(2.21)		
		Pb	(262)	(2.80)	(17.5)		(87.3)		
		Zn	(2369)	(5.98)	(36.5)		36.5		
		Fe	(39,315)	-	-		-		
24	Peninsular Malaysia (landfill; 2011–2012; 63 µm)	Cd	(3.90)	(4.70)	(39.0)	(8.43)	(1170)	1335	This study
		Cu	(451	(3.59)	(18.0)		(90.2)		
		Ni	(24.9)	(−0.59)	(0.44)		(2.22)		
		Pb	(168)	(2.16)	(11.2)		(56.0)		
		Zn	(1041)	(4.79)	(16.0)		(16.0)		
		Fe	(24,186)	-	-		-		
25	Peninsular Malaysia (mining; 2011–2012; 63 µm)	Cd	(2.54)	(4.08)	(25.4)	(4.86)	(762)	892	This study
		Cu	(517)	(3.79)	(20.7)		(103)		
		Ni	(19.3)	(−0.96)	(0.34)		(1.72)		
		Pb	(64.6)	(0.78)	(4.31)		(21.5)		
		Zn	(225)	(2.58)	(3.46)		(3.46)		
		Fe	(64,606)						
26	Peninsular Malaysia (plantation; 2011–2012; 63 µm)	Cd	(1.09)	(2.86)	(10.9)	(1.43)	(327)	348	This study
		Cu	(18.4)	(−1.03)	(0.74)		(3.68)		
		Ni	(13.9)	(−1.43)	(0.25)		(1.24)		
		Pb	(44.0)	(0.23)	(2.93)		(14.7)		
		Zn	(73.9)	(0.98)	(1.14)		(1.14)		
		Fe	(38,891)	-	-		-		
27	Peninsular Malaysia (residential; 2011–2012; 63 µm)	Cd	(0.83)	(2.47)	(8.30)	(1.37)	(249)	271	This study
		Cu	(31.3)	(−0.26)	(1.25)		(6.26)		
		Ni	(8.58)	(−2.13)	(0.15)		(0.77)		
		Pb	(37.5)	(0.00)	(2.50)		(12.5)		
		Zn	(145)	(1.95)	(2.23)		(2.23)		
		Fe	(30,738)	-	-		-		
28	Peninsular Malaysia (rubbish heap; 2011–2012; 63 µm)	Cd	(3.19)	(4.41)	(31.9)	(11.2)	(957)	1263	This study
		Cu	(821)	(4.45)	(32.8)		(164)		
		Ni	(26.3)	(−0.51)	(0.47)		(2.35)		
		Pb	(365)	(3.28)	(24.3)		(122)		
		Zn	(1114)	(4.89)	(17.1)		(17.1)		
		Fe	(26,039)	-	-		-		

Note: The cited metal data in the soils from the literature were recalculated for the values of I_geo_, CF, PLI, ER, and PERI by using the background metal concentrations (Cd: 0.10 mg/kg; Cu: 25.0 mg/kg; Ni: 56.0 mg/kg; Pb: 15.0 mg/kg; Zn: 65.0 mg/kg) in the earth’s upper continental crust (UCC) proposed by Wedepohl [48], and the toxic response factor (T_R_) of a single element (T_R_ =) (Cd: 30.0; Cu: 5.00; Ni: 5.00; Pb: 5.00; Zn: 1.00) according to Hakanson [65].

## Data Availability

Not applicable.
